# Heterosexist microaggressions, student academic experience and perception of campus climate: Findings from an Italian higher education context

**DOI:** 10.1371/journal.pone.0231580

**Published:** 2020-04-16

**Authors:** Anna Lisa Amodeo, Concetta Esposito, Dario Bacchini

**Affiliations:** 1 Department of Humanistic Studies, University of Naples Federico II, Naples, Italy; 2 SInAPSi Center (Services for Active and Participated Inclusion of Students), University of Naples Federico II, Naples, Italy; University of California Los Angeles, UNITED STATES

## Abstract

While overt instances of harassment and violence towards LGBQ+ individuals have decreased in recent years, subtler forms of heterosexism still shape the social and academic experience of students in higher education contexts. Such forms, defined as microaggressions, frequently include environmental slights that communicate hostile and derogatory messages about one’s sexual-minority status. However, there is some evidence suggesting that environmental microaggressions have deleterious effects on all students, regardless of their sexual orientation. The aim of the current study was to examine how heterosexist environmental microaggressions on campus contributed to heterosexual and non-heterosexual students’ negative perceptions of campus climate. We also analyzed whether the effect of microaggressions on campus climate was mediated by student social integration on campus. Data were collected in 2018 through an anonymous web-based survey that involved students from a large university of Southern Italy. The sample consisted of 471 students from 18 to 33 years old. Thirty-eight (8.1%) students self-identified as non-heterosexual. Measures included self-reported experiences of environmental microaggressions on campus, student degree of satisfaction with peer-group and student-faculty interactions, perceptions of faculty concern for student development, and of the overall campus climate. The structural equation model showed that heterosexist environmental microaggressions on campus were associated with negative perceptions of campus climate through lowered satisfaction with peer-group interactions and perceptions of faculty concern for student development, for both heterosexual and non-heterosexual students. Overall, the findings of this study suggest that heterosexist microaggressions within campus environments are negatively associated with students’ perceptions of campus climate, regardless of their sexual orientation. Both faculty and peers play an important role in creating an environment that supports the inclusivity of diversity and fosters a greater sense of belonging to the campus community.

## Introduction

The term campus climate is commonly used to describe how individuals and groups experience membership in the campus community. It reflects the inclusivity dynamics of the organization and the degree to which students, faculty, and staff feel included or excluded in the environment [1]. Critical incidences, harassment and bias, interaction between individuals and groups, and overall perceptions are all substantial indicators of campus climate [2]. A large body of research has highlighted that an inclusive and welcoming climate within academic contexts significantly impacts students’ academic progress and achievement and their level of satisfaction with their university [2]. However, this issue remains of crucial concern when examining campus climate for minority groups, such as lesbian, gay, bisexual, queer, and other sexual minority students (LGBQ+). Despite the increased visibility of LGBQ+ people on campuses during the last two decades [3], heterosexist and cisgender beliefs that people naturally engage only in relationships with people of the other sex and assume distinct gender roles, respectively, remain prevalent in educational settings. Recent research suggests that sexual and gender minorities tend to perceive the university campus climate as being more hostile and dangerous than do their non-LGBQ+ peers [4]. LGBQ+ university students often report overt experiences of intimidation, harassment, and violence in academic environments. They also describe social exclusion at both institutional and interpersonal level, along with subtle, although damaging, hostilities that take the form of heterosexist environmental microaggressions: actions that take place within the environment but are not directed at specific target, such as the telling of anti-LGB jokes that can be heard by anyone within earshot [5,6]. Both covert and overt experiences contribute to the development of a negative perception of campus climate [7], which may prevent LGBQ+ students from achieving academic success [8,9]. However, heterosexist dynamics that materialize in slurs, hate speech, and a climate of intolerance of diversity may impact all students, regardless of whether they personally identify as a member of the group being stigmatized [10].

The aim of the current study was to examine how being witness of heterosexist environmental microaggressions on campus is associated with student social integration (in terms of quality of interactions with peer and faculty and perception of faculty concern for student development) and contributes to develop negative perceptions of campus climate. More specifically, we investigated whether the association between microaggressions and perception of campus climate was mediated by their impact on specific dimensions of student social integration into the campus community. Finally, because not only sexual minority individuals may be targeted with microaggressions on campus, we examined these associations considering LGBQ+ as well as heterosexual students. We hypothesized that the impact of microaggressions will be significant in both groups, but stronger in the LGBQ+ group. Notably, while most of the literature on campus climate focuses on U.S. institutions, with some attention paid to Canadian and British contexts, this study is the first to focus on students’ experiences in an Italian higher education institution.

### Heterosexist microaggressions within higher education institutions

The discussion and investigation of microaggressions originated in the literature about racial and ethnic minorities [11], and only in the last decade have scholars started to investigate microaggressions against sexual minority groups [12–14]. The literature describes microaggressions as “the everyday verbal, nonverbal, and environmental slights, snubs, or insults, whether intentional or unintentional, that communicate hostile, derogatory, or negative messages to target persons based solely upon their marginalized group membership” [15]. A typical example of microaggression that targets sexual minority groups is using the word “gay” to describe someone, or something (referring to objects or situations), that is socially awkward. In this case, the message is that this person, activity, or thing is not valuable or enjoyable. Messages propelled by microaggressions based upon sexual orientation or gender identity support social structures based on exclusion [16] and reinforce the cultural message that violations of heteronormativity are unacceptable [17].

Heterosexist microaggressions are common on campuses [18,19] and appear to be even more prevalent than blatant hostility [20]. One possible explanation for this phenomenon is that, in comparison to blatantly hostile remarks, these manifestations are often dismissed as nondiscriminatory, not necessarily related to anti-gay prejudice, and considered harmless by those who use them [16,21]. Using the phrase “that’s so gay”, for instance, may be legitimized by contemporary slang use, such that its use is separated from the group that is its target [17].

Although research on the detrimental effects of microaggressions based upon sexual orientation is still underdeveloped, with most of the studies using a qualitative approach [13,22], it would be difficult to argue that these kinds of overt forms of discrimination and aggression have no consequences on the health of sexual minorities. As theorized within the minority stress model [23], individuals from marginalized groups experience chronic stress due to stigma, prejudice, and discrimination that shapes their life environments. This chronic stress put them at risk for poor mental and physical health. Overall, the studies that have explored the effects of microaggressions on LGBQ+ students health appear to support the minority stress model. The 2017 National School Climate Survey reported that 91.8% of LGBQ+ students in K-12 and college who heard “gay” used in a negative way (e.g., “that’s so gay”) felt distressed because of this language [24]. In a study by Woodford and colleagues, whose aim was to evaluate the social and physical well-being of LGB college students who reported hearing the phrase “that’s so gay” [21], 87% of the respondents reported hearing that phrase at least once over the past year. This experience was associated with a high frequency of headaches, poor eating or trouble with appetite, and feeling left out at the university. Other similar studies found that microaggressions targeting sexual minority students on campus were positively related to psychological distress in LGBQ+ college students [19,25], independent from experiences of direct victimization.

### Microaggressions and student academic experiences

Previous studies reported that heterosexist microaggressions create a hostile climate for LGBQ+ individuals and groups [7]. Furthermore, encountering discrimination on campus can interfere with the academic development of sexual minority students [26]. In fact, due to the psychological stress associated with discrimination [20], LGBQ+ students may withdraw, both psychologically and physically, from their institution, and thus develop negative interactions on campus, damaging perceptions of the overall academic experience, and negative overall perceptions of campus climate [27]. However, how heterosexist microaggressions contribute to negative academic development among sexual minority and heterosexual students remains under-researched [27].

Academic development is generally considered to be a multifaceted construct that involves both academic (in terms of student’s academic performance and his or her level of intellectual development, commitments to the institution, and goals associated with graduation and career) and social integration components [28]. Past work on heterosexism evidenced that environmental experiences of discrimination may interfere with students’ academic functioning [5,10,26,27], paying most attention to dimensions of academic integration and underscoring the investigation of students’ social integration within the academia, including the quality of peer-group interactions and student interactions with faculty, along with perceptions of the faculty’s concern for student development. Mathies and colleagues [27], for instance, analyzed data from 574 LGBQ+ college students and reported that hearing heterosexist phrases on campus was significantly associated with academic stress among sexual minority students, including difficulty in meeting academic standards, finding courses too demanding, and dissatisfaction with performance developmental challenge. In the study by Woodford and Kulick [26], which included 381 sexual minority college students, heterosexism on campus was associated with lower academic disengagement, lower grade point average, lower institutional satisfaction, and lower social acceptance for sexual minority students. To date, only a few researchers have examined the impact of heterosexist discrimination on social integration dimensions, also analyzing the impact of this discrimination on all students, regardless of their sexual orientation [5,10]. In a sample of 3,128 college students, Silverschanz and colleagues [5] found that, although sexual minority students were more likely to report experiences with heterosexism, 39% of heterosexual students reported hearing environmental heterosexist hostility (e.g., hearing others make offensive remarks or jokes about gay people, or call someone homophobic names). Furthermore, they found that individuals who experienced discrimination were more likely to report poor mental health (anxiety, depression) and academic outcomes (e.g., social acceptance, instructor relations, school avoidance), independent of their sexual orientation. Similarly, Norris and colleagues [10] found that these environmental microaggressions significantly and negatively predicted perceptions of personal safety, belongingness, and teacher connectedness in heterosexual and non-heterosexual students. There are several reasons that could explain why heterosexist microaggressions may impact heterosexual students’ social integration on campus. First, heterosexual students may be directly targeted with microaggressions, based on a presumption of sexual-minority status. Otherwise, the effects of heterosexist microaggressions could be related to students’ relationships with members of the stigmatized group, which would make them more sensitive to discrimination on campus [10]. However, even if heterosexual students are not directly connected to the LGBQ+ community, hearing discriminatory speech or perceiving negativity toward a specific group might interfere with students’ comfort with campus climate, Indeed, seeing minority groups individuals treated as second class citizens, and not as valued members of the campus community, might lead them to believe that they will be treated in the same way when needed [10].

### Heterosexism in the Italian context

Stereotypical gender roles are more prominent in Italy than in other Western regions [29]. This is mainly due to the relatively high influence that the Catholic Church has in shaping Italian beliefs and attitudes toward homosexuality and deviations from conventional gender roles. Other influences come from the Mediterranean traditions that are deeply rooted through the country, especially in the Southern, where the current study was conducted, which emphasize the role of honor and shame in shaping masculine and feminine socialization [29]. As a consequence, the recognition of civil rights for homosexual people has progressed and still moves slower in Italy with respect to other Western countries. Some important changes have occurred during the last decade as a part of Italy’s efforts to strengthen measures to contrast discrimination and violence based on sexual orientation and gender identity. Same-sex civil unions and unregistered cohabitation were legally recognized on June 2016 and, under the European Union direction, legislation prohibiting discrimination based on sexual orientation and gender identity in the field of both public and private employment has been introduced. Furthermore, in April 2013 a National Strategy to prevent and contrast discrimination on grounds of sexual orientation and gender identity (2013–2015) was adopted by a Ministerial Decree, but it wasn’t renewed after 2015, confirming the lack of proper legislation, at both national and local level, to protect and ensure equal rights for sexual minorities living in Italy [30].

Overall, previous studies have found that Italian sexual minority people frequently face several heterosexist prejudices in their daily life [31]. Research about negative attitudes toward homosexuality in Italy, although limited, supports the hypothesis that Italian people are quite ambivalent toward homosexuality [32,33]. On the one hand, homosexuality is considered as a sin or a deviation from normal development; on the other hand, it is considered as a private matter, something that should be neither persecuted nor protected by law. Consistent with the Italian gender role tradition, negative attitudes are more prominent toward gay men compared with lesbians, supporting the representation of male homosexuality as a visible threat to the traditional gender norms, and the idea that only the traditional gender-conforming, heterosexual behavior is socially acceptable [34].

### The present study

**Fig 1:**
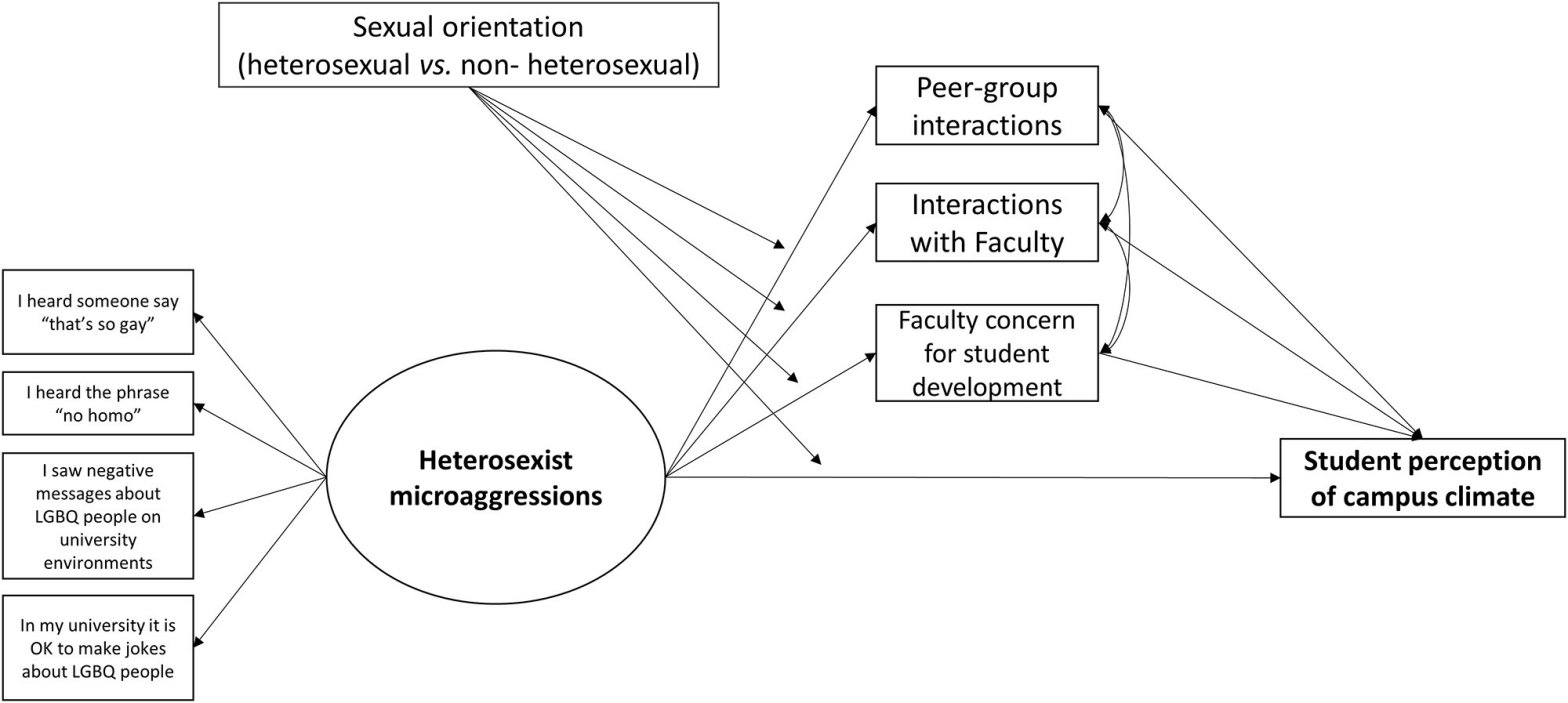
The hypothesized model.

This study sought to expand on previous knowledge by investigating social integration pathways that link heterosexist microaggressions on campus with student perception of campus climate. More specifically, we hypothesized that witnessing environmental microaggressions will decrease student social integration and the overall perception of campus climate, based on previous literature highlighting that psychological stress associated with discrimination leads students to develop negative interactions on campus and damaging perceptions of the overall academic experience (Hypothesis 1: main effects). Furthermore, we hypothesized that microaggressions are associated with perception of campus climate through their negative effect on student social integration (Hypothesis 2: mediation effects). Finally, we tested whether both the direct and indirect effect of microaggressions on perceptions of campus climate are stronger for non-heterosexual compared to heterosexual students, because of their being directly targeted with them (Hypothesis 3: moderation and moderated mediation effects). The hypothesized model is depicted in Fig 1. As previous research suggested that perceptions of a negative climate due to experiences of heterosexist microaggressions may vary by gender identity, with heterosexual women perceiving a more negative climate for sexual minorities than men [17], we controlled all the effects for gender identity, age, academic class rank, and specialized academic program were also included in the study as control variables.

## Materials and methods

### Participants and procedure

Data were collected in 2018 through an anonymous web-based survey of students from a large university of Southern Italy. The study was designed to conform to the principles of the Declaration of Helsinki on Ethical Principles for Medical Research Involving Human Subjects and was approved by the University Institutional Review Board. Students were invited to complete the survey by faculty engaged in the institutional committee for Diversity, Equity, and Inclusion, who were contacted by staff working at the Anti-discrimination division of the University Service Center (SInAPSi–Center for Active and Participated Inclusion of Students). They were asked to share the survey with any other students they knew at the same university, a strategy that led to a snowball sampling recruitment procedure. Privacy was guaranteed to participants in accordance with Italian laws 196/2003 and 101/2018. Informed consent was obtained prior to beginning data collection. The participation was voluntary, and participants could withdraw at any time without any adverse consequence. No survey questions were mandatory.

Eighteen surveys were excluded from analysis due to missing data on all key study variables. The final sample comprised 471 Italian students (65% female). Participants were 18 to 33 years old (*M* = 22.93, *SD* = 3.11). They were enrolled in both humanistic (56%) and scientific (44%) academic programs. No participant self-identified as transgender or transsexual. Thirty-eight (8.1%) students self-identified as non-heterosexual: 21 students self-identified as bisexual (17 females and 4 males), 6 as gay, 6 as lesbian; 5 female students self-identified as pansexual and questioning (3 and 2, respectively). Due to the limited number of participants in each sexual orientation category, we created a dummy variable (i.e., heterosexual vs. non-heterosexual) to use in the study’s analyses. Descriptive statistics of sample characteristics are reported in Table 1.

**Table 1:** Sample characteristics.

Characteristics	Total (N = 471) N (%) or *M* (*SD)*	Heterosexuals (n = 433) N (%) or *M* (*SD)*	Non-heterosexuals (n = 38) N (%) or *M* (*SD)*
Gender			
Female	307 (65.2)	279 (64.4)	28 (73.7)
Male	164 (34.8)	154 (35.6)	10 (26.3)
Age	22.82 (2.67)	22.82 (2.63)	22.82 (3.14)
Class rank			
Freshman year	67 (14.3)	60 (13.9)	7 (18.4)
Other	404 (85.7)	373 (86.1)	31 (81.6)
Specialized academic program			
Scientific	205 (43.5)	193 (44.6)	12 (31.6)
Humanistic	266 (56.5)	240 (55.4)	26 (68.4)

*M* = mean; *SD* = standard deviation.

### Measures

#### Sociodemographic characteristics and controls

Sociodemographic variables included sex assigned at birth (male, female, intersex), actual perceived gender (man, woman, and other with specification required), sexual orientation (lesbian, gay, bisexual, asexual, and other with specification required), and age. In terms of academic variables, we asked participants to indicate the specialized area of academic course (scientific vs. humanistic) and the academic class rank, to distinguish first-year students from all other students (freshman vs. other).

#### Heterosexist microaggressions

To assess the frequency of heterosexist microaggressions on campus, we used four items based on the LGBQ Environmental Microaggression Scale developed by Woodford et al. [6]: I heard someone say “that’s so gay” to describe something as negative, stupid, or uncool; I heard the phrase “no homo”; I saw negative messages about LGBQ people on university environments or social media (e.g., Facebook, Twitter); In my university it is OK to make jokes about LGBQ people. Respondents were asked to indicate the frequency of each incident on campus over the past year or since being a university student (if less than 1 year). Response options ranged from “never” (1) to “very frequently” (5). The analysis of the scale factor structure showed an adequate fit to the data, χ^2^ (2) = 6.79, *p* = .05, comparative fit index (CFI) = .99, root mean square error of approximation (RMSEA) = .07 90% confidence interval (C.I.) [.01, .11], standardized root mean square residual (SRMR) = .02. Cronbach’s alpha was .75.

#### Social integration

Social integration was measured through items extracted from the Institutional Integration Scale developed by Pascarella and Terenzini [28]. Social integration was divided into three subscales: peer-group interactions (seven items, sample item: Since coming to this university, I have developed close personal relationships with other students; Cronbach’s alpha = .85) and interactions with faculty (five items, sample item: I am satisfied with the opportunities to meet and interact informally with faculty members; Cronbach’s alpha = .91), which measure the quality of peer-group interactions and of student interactions with faculty, respectively; faculty concern for student development and teaching (four items; sample item: Most of the faculty I have had contact with are interested in helping students growth in more than just academic area; Cronbach’s alpha = .80). The measures were translated from English into Italian by two native Italian speakers, experts in psychology and fluent in English. Two different versions were obtained and compared, achieving a final agreement. Then, an American native English speaker translated the obtained version from Italian to English to confirm that translation was accurate. All items were rated on a scale from strongly disagree (1) to strongly agree (5), with higher values indicative of greater positive perceptions of social integration. The confirmatory factor analysis confirmed the psychometric structure of the scale, χ^2^ (98) = 463.80, *p* < .001, CFI = .93, RMSEA = .08, SRMR = .05.

#### Perceptions of campus climate

To assess campus climate, we used questions from Rankin’s Campus Climate Survey [18]. Using a scale from 1 to 5, participants were asked to rate the overall university climate on a series of five dimensions: hostile/friendly, excluding/including, regressing/improving, not welcoming/welcoming, and disrespectful/respectful. The mean average score was used as a measure of the overall campus climate as perceived by students (Cronbach’s alpha = .78).

### Statistical analyses

The study’s hypotheses were tested using structural equation modeling (SEM) in Mplus version 8 [35]. The percentage of missing values for each measure was low (it was either zero or less than 1%). Little’s Test [36] of missing data was not statistically significant, χ2(52) = 34.57, p = .97, suggesting the missingness on one variable was unrelated to the other measured or unmeasured variables. Accordingly, full information maximum-likelihood (FIML) was used to handle missing data [35]. Values of skewness and kurtosis for variables did not substantially deviate from a normal distribution, and thus analyses were performed by using the Maximum Likelihood (ML) estimator. One latent factor of heterosexist microaggressions was estimated as part of the main structural equation model. Several confounding variables were considered in the study: actual perceived gender (man vs. woman), age, specialized area of academic course (scientific vs. humanistic), and the academic class rank (freshman year vs. other). Multiple fit indices were used to evaluate model fit: chi-square likelihood ratio statistic, CFI, RMSEA with associated 90% C.I., and SRMR. Guided by suggestions provided in Hu and Bentler [37], an acceptable model fit was defined by the following criteria: CFI (≥ .95). RMSEA (≤ .06, 90% C.I. ≤ .06), and SRMR (≤ .08).

To address our first hypothesis, we initially tested the main effects of heterosexist microaggressions on dimensions of social integration and overall perception of campus climate (Hypothesis 1). In this first step, sexual orientation was considered to be a predictor of academic and social integration and perceptions of university climate, along with heterosexist microaggressions. Then, we examined whether each dimension of social integration mediated the relationship between heterosexist microaggressions and campus climate (Hypothesis 2). The mediation effects were tested using bias-corrected bootstrap confidence intervals based on 5000 resamples. Confidence intervals that do not contain zero indicate a significant indirect effect via the specific mediator. Next, we tested the moderating effect of sexual orientation in the relationships between heterosexist microaggressions and campus climate (i.e., conditional direct effect) and between heterosexist microaggressions and dimensions of social integration (conditional indirect effect; Hypothesis 3). Significant conditional effects were probed using the pick-a-point approach [38].

## Results

### Descriptive statistics and bivariate correlations

Means, standard deviations, and Pearson bivariate correlations between all variables are shown in Table 2. The results highlighted a negative correlation between heterosexist microaggressions and faculty concern for student development and teaching in both heterosexual and non-heterosexual students. Perception of campus climate was negatively associated with heterosexist microaggressions and positively associated with social integration. Among the control variables, age was significantly and negatively associated with campus climate in non-heterosexual students. Heterosexual women, compared to men, scored lower on heterosexist microaggression. Also, heterosexual freshman students rated their interactions with faculty and faculty concern for student development and teaching more satisfying compared to students in other years. Specialized academic program had no significant associations with any variable in the study, thus we decided to remove it from further analyses.

**Table 2:** Descriptive statistics and bivariate correlations among study’s variables.

	1	2	3	4	5	6	7	8	9	Mean	*SD*
1. Heterosexist Microaggressions (latent variable)	1	.01	-.02	-.32*	-.62***	.24	-.16	.11	-.05	0 (.12)	.93 (.19)
2. Peer-group interactions	-.18***	1	.31*	.43***	.33*	.21	-.26	.08	-.13	3.59 (3.35)	.82 (1.02)
3. Interactions with faculty	-.05	.22***	1	.53***	.27*	.02	-.04	.06	-.17	2.71 (2.77)	1.09 (1.09)
4. Faculty concern for student development and teaching	-.19***	.25***	.46***	1	.41***	.06	-.08	-.07	-.10	2.98 (2.95)	.95 (.79)
5. Campus climate	-.17***	.40***	.25***	.38***	1	-.33*	-.04	-.12	-.08	3.34 (3.27)	.74 (.72)
*Control variables*											
6. Age	.07	-.08	.05	-.09	-.09	1	.07	-.18	.09		
7. Gender identity (women)	-.11*	.06	.03	.05	.05	-.05	1	.02	.35*		
8. Class rank (other)	.02	-.06	-.10*	-.09*	-.01	.13*	.01	1	.25		
9. Specialized academic program (humanistic area)	.01	-.02	-.02	-.02	.03	-.04	.12**	.14**	1		

SD = standard deviation. Means and standard deviations that refer to non-heterosexual students are reported in brackets. Values for heterosexual students are below the diagonal. Values for non-heterosexual students are above the diagonal.

***p < .001;

**p < .01;

*p < .05;

^†^p < .10.

### Frequency of heterosexist microaggressions on campus

Most participants (66.6%) reported “occasionally to very frequently” being witness to heterosexist microaggressions on campus. Percentages were similar in heterosexual (66.7%) and non-heterosexual students (65.8%; χ^2^ (2) = .01, *p* = .52). The most common microaggression reported by participants was hearing someone say “that’s so gay” (58.2%).

### Structural equation modeling

**Fig 2:**
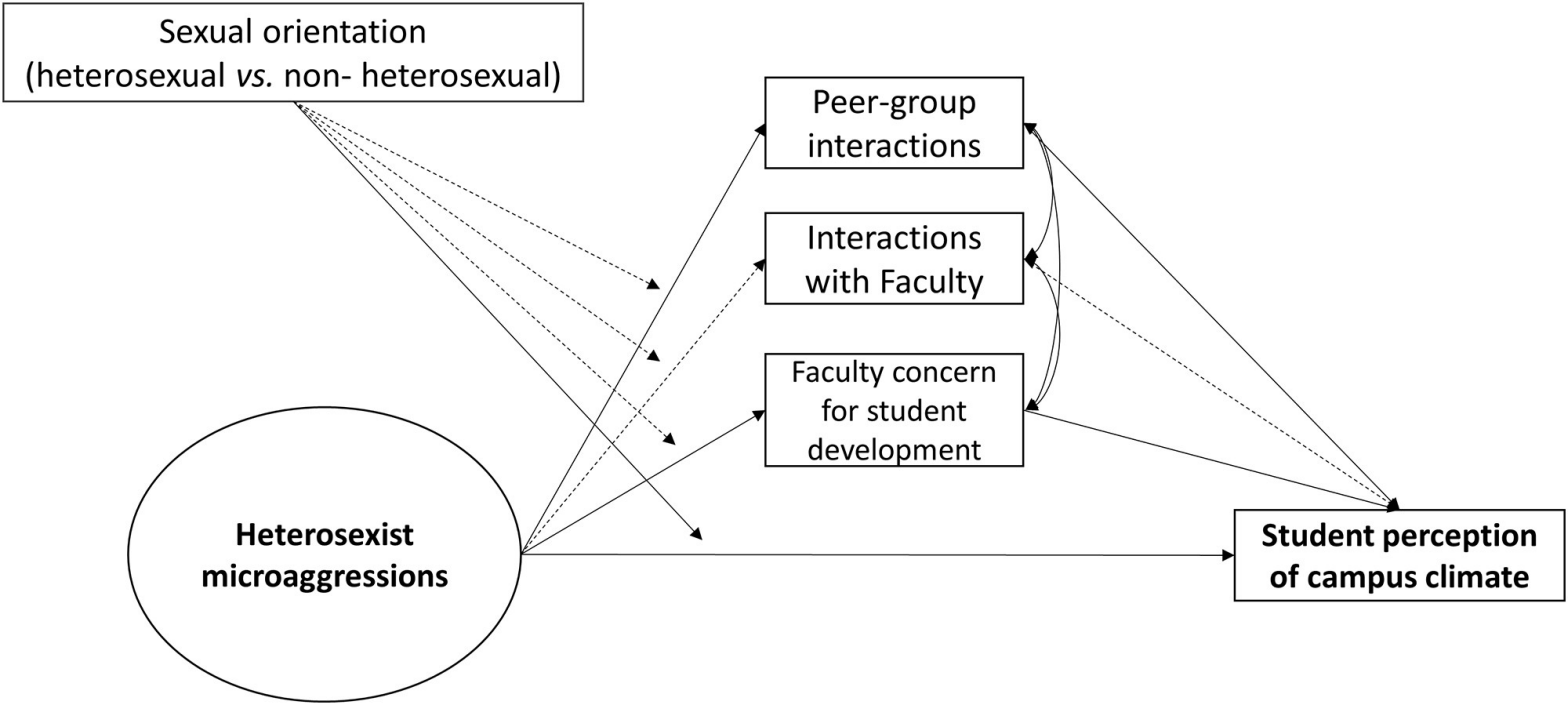
Significant and non-significant paths from the tested model. Solid lines indicate significant paths. Dashed lines represent non-significant paths.

**Fig 3:**
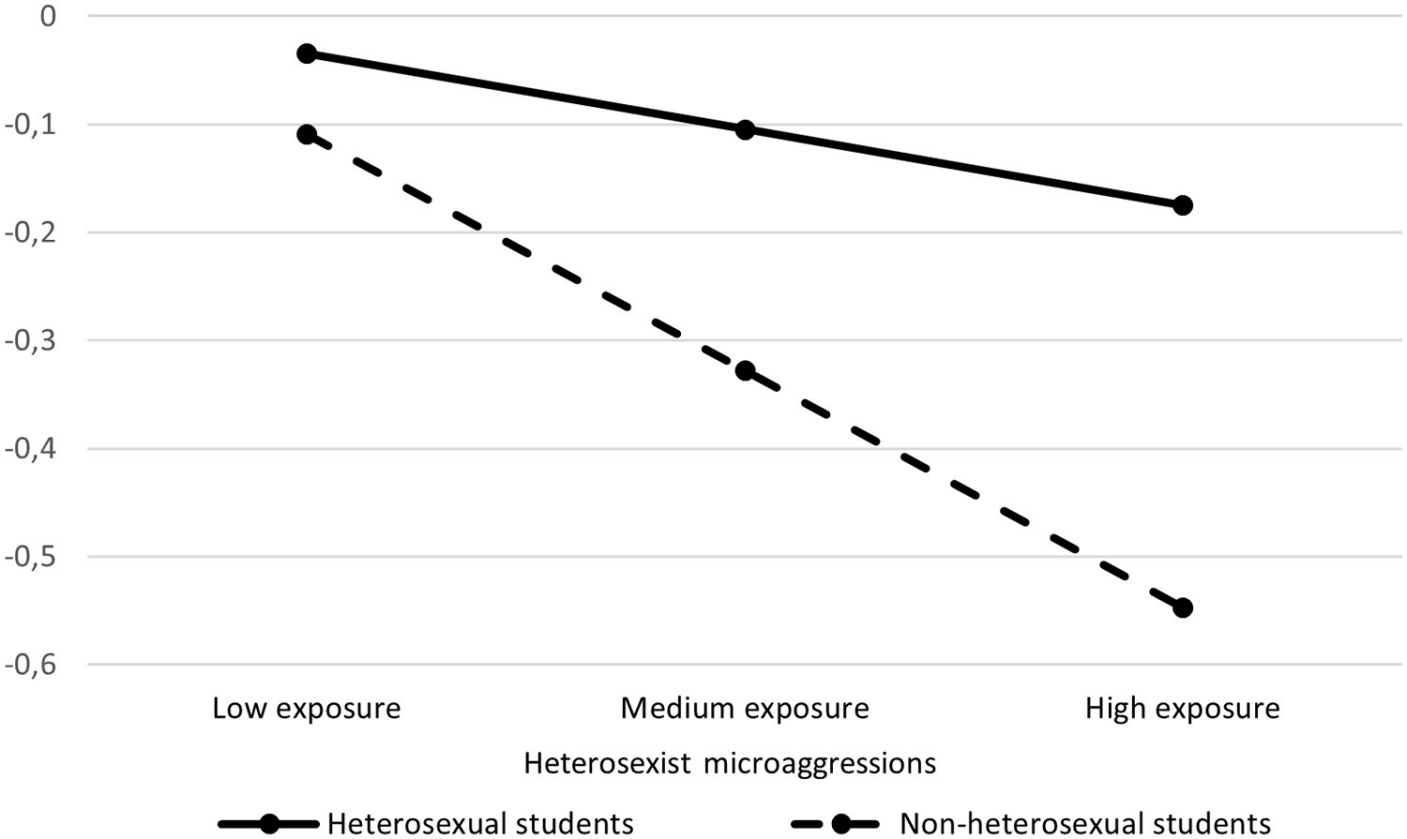
The direct effect of heterosexist environmental microaggressions on student perception of campus climate, conditional on student sexual orientation. The effect was significant for non-heterosexual students, b = -.42, *p* < .001, bootstrap C.I. [-.69, -.14], and non-significant for heterosexual students, b = -.04, *p* = .26, bootstrap C.I. [-.13, .03].

Results of the SEM are presented in Table 3 and displayed in Fig 2. The hypothesized model showed an adequate fit to the data (χ^2^ (27) = 26.69, *p* = .48, CFI = 1.00, RMSEA = .00 with 90% C.I. [.00, .03], SRMR = .03). Heterosexist microaggressions were significantly and negatively associated with peer-group interactions and faculty concern. High quality peer-group interactions and faculty concern were associated with a positive student perception of campus climate. There was only a marginal significant effect that linked microaggressions with negative perceptions of campus climate (*p* = .06). Sexual orientation was not significantly associated with social integration dimensions or perceptions of campus climate. The mediation analysis highlighted two significant indirect effects: Heterosexist microaggressions were associated with negative perceptions of university climate through the decrease of interactions quality with peer (β = -.05, *p* = .001, 95% bootstrap C.I. [-.08, -.02]) and the faculty concern for student development and teaching (β = -.05, *p* = .001, 95% bootstrap C.I. [-.08, -.02]). The model explained 26% of the variance in campus climate perception (*p* = .001). Finally, the moderation analysis evidenced that only the direct association between heterosexist microaggressions and perceptions of university climate was moderated by sexual orientation (interaction term: β = -.13, *p <* .001). The analysis of simple slopes indicated that only non-heterosexual students showed a decline in perceptions of university climate when exposed to a high frequency of heterosexist microaggressions (b = -.42, *p* < .001; Fig 3).

**Table 3:** Standardized parameters resulting from structural equation modeling.

Focal variables	Mediators			Dependent Variable
	Peer-group interactions	Interactions with faculty	Faculty concern for student development and teaching	Campus climate
	β [90% CI]	β [90% CI]	β [90% CI]	β [90% CI]
Heterosexist Microaggressions (latent variable)	-.16*** [-.26, -.06]	-.04 [-.15, .08]	-.19** [-.28, -.10]	-.09^†^ [-.19, .00]
Peer-group interactions				22*** [.21, .38]
Interactions with faculty				-.02 [-.05, .14]
Faculty concern for student development and teaching				.21*** [.17, .35]
Sexual orientation (Non-heterosexual)	-.07 [-.20, .03]	.03 [-.07, .11]	-.02 [-.10, .06]	.01 [-.06, .09]
*Control variables*				
Age	-.03 [-.14, .07]	.07 [-.03, .18]	-.05 [-.14, .06]	-.05 [-.15, .02]
Gender identity (Women)	.01 [-.08, .10]	.03 [-.07, .11]	.02 [-.07, .11]	.01 [-.06, .11]
Class rank (Other)	-.04 [-.12, .04]	-.10* [-.18, -.01]	-.09^†^ [-.18, .00]	.02 [-.05, .12]

***p < .001;

**p < .01;

*p < .05;

^†^*p* < .10.

## Discussion

While the rights of sexual minority students have significantly progressed over time, especially in terms of protection against overt aggression and discrimination, promotion of LGBQ+ integration on campuses remains an important challenge for higher education institutions. Recent work has documented examples of heterosexist dynamics that shape the experience of LGBQ+ and heterosexual university students on campuses. These dynamics materialize in so-called microaggressions that include overheard phrases such as “that’s so gay” [21], gay jokes, and other slurs [5] that might affect sexual minorities and non-heterosexual health, as well as their academic outcomes and general satisfaction with the institution. The frequency of heterosexist environmental microaggressions among university students reported in this study was consistent with previous research [10] that reported a prevalence estimate of approximately 50% and 70% in heterosexual and non-heterosexual students, respectively.

The aim of this study was to examine heterosexual and non-heterosexual students’ experiences of heterosexist microaggressions on campus and how these experiences impact the students’ social integration and overall perceptions of campus climate. More specifically, we hypothesized that experiences of microaggressions decrease student social integration and the overall perception of campus climate (Hypothesis 1: main effects). Furthermore, we hypothesized that microaggressions are associated with a negative perception of campus climate through their deleterious effect on student social integration (Hypothesis 2: mediation effects). Finally, we tested whether both the direct and indirect effect of microaggressions on perceptions of campus climate are stronger for non-heterosexual compared to heterosexual students (Hypothesis 3: moderation and moderated mediation effects).

Consistent with our hypotheses, the results showed that being exposed to heterosexist microaggressions on campus was associated with lower levels of social integration, specifically in terms of quality of peer-group interactions and perceptions of faculty concern for student development (Hypothesis 1). Furthermore, we found that microaggressions indirectly impacted the overall perception of campus climate by decreasing the quality of peer-group interactions and lowering the perception of faculty concern for student development (Hypothesis 2) in heterosexuals and non-heterosexuals. However, in non-heterosexual students, heterosexist microaggressions also directly affected the development of negative perceptions about campus climate (Hypothesis 3).

With respect to our first hypothesis, we found significant associations of microaggressions with student social integration within the campus community. This is consistent with previous studies that linked heterosexist microaggressions on campus with student academic outcomes [10,27]. As concerns sexual minority students, one plausible explanation for this result is the association of microaggressions with psychological distress [19], which in turn would relate to academic outcomes [39]. The hypothesis of psychological distress is well supported by the minority stress theory [23], according to which increased exposure to heterosexist microaggressions might lead LGBQ+ students to experience chronic stress. This encounter would in turn undermine LGBQ+ students’ sense of belonging to their academic’s community and contribute to poor academic outcomes. Furthermore, heterosexism can also undermine students’ mental health and self-esteem, thereby presenting another barrier to academic achievement [40]. With respect to heterosexual students, several hypotheses have been advanced for explaining why heterosexist microaggressions may impact heterosexual students’ social integration on campus. Norris and colleagues [10], for example, found that knowing an openly LGBT student amplified the risk, for heterosexual students, of lowered sense of belongingness on campus after a repeated experience of hearing other students make slurs. However, the authors concluded that hearing discriminatory speech or perceiving the climate to be intolerant to a specific group might interfere with students’ social integration within the campus community, regardless of their directly connection to the LGBQ+ community. Another potential explanation for this phenomenon is that seeing peers, faculty, and the overall institution staff not treat minority group individuals as valued members of the campus community may lead heterosexual students to believe that they in turn will be treated in the same way and will not receive support when needed [10]. A novel finding in our study is that heterosexist microaggressions within university environments were associated with specific dimensions of social integration. This factor reflected the quality of students’ interactions with peers and students’ perception that faculty are concerned about students’ development. There was no significant association between environmental microaggressions and students’ daily interactions with faculty. These differential effects suggest that heterosexist microaggressions within university environments are interpreted by students as something that involves interpersonal relationships, in terms of horizontal relationships with peers. The role of faculty would be crucial to the extent that, if microaggressions occur, they may be perceived by students as a consequence of a lack of faculty empathy and concern for students’ well-being and development—perhaps due to a passive response from faculty in addressing and facing the problem—that reflects the message that denigration is normal. This conjecture is in line with previous research on homophobic bullying at school, which suggested that teachers play an important role in creating and maintaining a supportive and inclusive learning environment [41]. Kosciw and colleagues [24], for instance, showed that LGBT students experienced less harassment and assault and better educational outcomes when their teachers intervened in incidents of homophobic bullying. In our study, interactions with faculty were not impacted by environmental microaggressions, perhaps because students experience these faculty interactions as strictly related to addressing the educational mission of colleges and universities, namely teaching. This interpretation is somewhat consistent with Mathies and colleagues’ study [27], which reported that microaggressions like “That’s so gay” and “No homo” were associated with academic stress but not with students’ satisfaction about their academic and intellectual experience at campus.

Partially consistent with our second hypothesis, we found that heterosexist microaggressions on campus indirectly contributed to a negative perception of campus climate through the negative effect they had on social integration in both heterosexual and non-heterosexual students. Specifically, episodes of heterosexist microaggressions on campus were associated with students’ reports of low quality peer-group interactions and low perception of faculty concern for student development that in turn contributed to negative overall perceptions of campus climate. This finding suggests that students’ perceptions of campus climate for diversity are not tied as strongly to experiences related to classes and studying, but, instead, they seem to depend on the way students experience relationships on campus. This is consistent with the study by Tetreault and colleagues [7], who stressed the importance of factors related to students’ daily lives (e.g., how they are treated in class, the support they receive from their friends), above and beyond the institution’s demographic profile, in determining a more positive perception of the environment.

Contrary to our third hypothesis, we did not identify that non-heterosexual students showed worse consequences than heterosexuals. However, while non-minority students, that are not directly targeted with heterosexist microaggressions, displayed a hostile perception of campus climate depending on the impact that those subtle forms of discrimination had on the quality of their social integration on campus, microaggressions directly contributed to the non-heterosexual students’ negative perceptions of campus climate, without necessarily impacting their social integration on campus. One possible explanation for this could be related to the fact that heterosexist microaggressions directly target sexual minority students, thus negatively impacting on their perception of campus climate, described as not welcoming and inclusive [5,7,10].

### Implications

Examining the campus climate constitutes an important part of the agenda for higher education institutions, especially in an era when evidence-based practices are strongly recommended in order to achieve ever-higher levels of educational and organizational performance. Differently from US contexts, where assessing campus climate is a consolidated tradition [18], in Italy and in Europe in general, this still remains a challenge. Noteworthy, however, is the great interest of the European Commission in promoting the development and the implementation of innovative methods and practices to foster inclusive learning environments. Within this framework, a consortium of 7 partners from 5 European countries (Italy, Ireland, Slovenia, Greece and Spain) is currently working on the development of a tool (namely, the XENIA Index) that will assist European universities in measuring how inclusive they are and in learning ways to shape the educational experience to be more welcoming and respectful of sexual minorities.

Overall, the results from the current study shed light on the importance of considering subtle forms of aggression and discrimination when dealing with issues of sexual minority inclusivity within campuses. The faculty response to microaggressions on campus appears to play a key role in creating negative perceptions of campus climate for heterosexual and non-heterosexual students. It appears to be crucial for institutions and faculty to consider how they can work to promote and foster a sense of support and respect for all students; these actions improve both heterosexual and sexual minority students’ well-being on campus. Along with enforcing universities’ anti-discrimination policies, including microaggressions as subtle, but harmful forms of discriminations, efforts should be made to increase faculty awareness about the nature of heterosexist environmental microaggressions and their negative consequences on student academic integration. This awareness will allow faculty to address microaggressions when they happen, during class discussions, or in other contexts. Peers can also serve a critical role, and they should be encouraged to intervene when they witness heterosexist microaggressions on campus. This action will contribute to the creation of an environment that supports the inclusivity of diversity and fosters a greater sense of belonging to the campus community for all students.

### Limitations

Several limitations of our study must be acknowledged. First, data were collected in a small sample of students, all coming from one higher education institution in Italy. While dealing with campus climate for sexual minorities in Italy represents an important novelty in the scientific literature, a more robust investigation that involves large student samples from several universities in Italy is required for the generalization of results. A further sample-related limitation concerns the limited number of LGBQ+ participants, that may have contributed to non-significant effects in the study. Furthermore, the results may have been contaminated by shared variance associated with the use of only students’ self-reports as a measurement method. Also, given the use of a cross-sectional design, we are unable to determine causal relationships between the study’s variables. Moreover, it is important to note that, while significant, only a limited percentage of campus climate variance was explained by predictors that were considered in this study. A potential explanation for this lower amount of explained variance could be related to the fact that the current study only focused on how frequently students witnessed environmental heterosexist microaggressions on campus, without considering whether microaggressions were perpetrated by peers, faculty, or staff. As reported by Norris and colleagues [10], although students are more likely to hear other students make slurs, faculty and staff also engage in discriminatory behaviors and this factor requires additional investigation. Furthermore, we only examined the effects of students’ passive witnessing of heterosexist microaggressions on campus, without considering possible effects of being directly targeted or victimized, as well as of actively contributing to heterosexist microaggressions. Future research should also investigate the possible differential impact of heterosexist microaggressions among specific sexual minority sub-groups, including transgender and bisexual people. Some studies, for instance, have highlighted that bisexual individuals experience additional stressors related to their sexual identity compared to lesbians and gay men. In fact, bisexuality is often viewed as an illegitimate and unstable sexual orientation, even by other sexual minorities. They can be perceived as sexually irresponsible, promiscuous or unable to have monogamous relationships [42].

## Conclusions

Higher education institutions are complex social systems where relationships among individuals and groups have a profound effect on the academic community’s ability to excel in teaching, research, and scholarship [3]. The results of this study support the importance of addressing subtle forms of heterosexist discrimination on campus in order to create a diverse and inclusive campus community, where not only sexual minorities are actively accepted, but all students feel equally welcome and engaged.

## Supporting information

S1 Data (XLSX)
